# Interventions to improve cultural competency in healthcare: a systematic review of reviews

**DOI:** 10.1186/1472-6963-14-99

**Published:** 2014-03-03

**Authors:** Mandy Truong, Yin Paradies, Naomi Priest

**Affiliations:** 1 McCaughey VicHealth Centre for Community Wellbeing, Melbourne School of Population and Global Health, The University of Melbourne, Carlton, Australia; 2 Centre for Citizenship and Globalization, Faculty of Arts and Education, Deakin University, Burwood, Australia

**Keywords:** Cultural competency, Healthcare, Health outcomes, Health disparities, Minority health, Systematic review

## Abstract

**Background:**

Cultural competency is a recognized and popular approach to improving the provision of health care to racial/ethnic minority groups in the community with the aim of reducing racial/ethnic health disparities. The aim of this systematic review of reviews is to gather and synthesize existing reviews of studies in the field to form a comprehensive understanding of the current evidence base that can guide future interventions and research in the area.

**Methods:**

A systematic review of review articles published between January 2000 and June 2012 was conducted. Electronic databases (including Medline, Cinahl and PsycINFO), reference lists of articles, and key websites were searched. Reviews of cultural competency in health settings only were included. Each review was critically appraised by two authors using a study appraisal tool and were given a quality assessment rating of weak, moderate or strong.

**Results:**

Nineteen published reviews were identified. Reviews consisted of between 5 and 38 studies, included a variety of health care settings/contexts and a range of study types. There were three main categories of study outcomes: patient-related outcomes, provider-related outcomes, and health service access and utilization outcomes. The majority of reviews found moderate evidence of improvement in provider outcomes and health care access and utilization outcomes but weaker evidence for improvements in patient/client outcomes.

**Conclusion:**

This review of reviews indicates that there is some evidence that interventions to improve cultural competency can improve patient/client health outcomes. However, a lack of methodological rigor is common amongst the studies included in reviews and many of the studies rely on self-report, which is subject to a range of biases, while objective evidence of intervention effectiveness was rare. Future research should measure both healthcare provider and patient/client health outcomes, consider organizational factors, and utilize more rigorous study designs.

## Background

Cultural competency is a broad concept used to describe a variety of interventions that aim to improve the accessibility and effectiveness of health care services for people from racial/ethnic minorities. It developed largely in response to the recognition that cultural and linguistic barriers between healthcare providers and patients could affect the quality of healthcare delivery. The targeted groups were mainly immigrant populations from non-English speaking countries with limited exposure to Western cultural norms [1].

Since its introduction in the 1980s, the range of cultural competency frameworks and models has burgeoned. Many models include dimensions of knowledge (e.g., understanding the meaning of culture and its importance to healthcare delivery), attitudes (e.g., having respect for variations in cultural norms) and skills (e.g., eliciting patients’ explanatory models of illness) [1]. Over time, the scope of cultural competency expanded beyond the interpersonal domain of the practitioner-patient/client interaction to include organizational and systemic cultural competency. Although the most often cited definition of cultural competency is that of Cross and colleagues [2], there is no one widely accepted and definitive conceptual cultural competency framework. The literature contains many analogous terms/concepts (e.g. culturally appropriate care, multicultural education) that add to the lack of clarity in this field.

There is an abundance of international literature related to cultural competency and the importance of its integration into all levels of health care. In the United States, the prominence of cultural competency within health policy and practice is largely attributed to federal and state regulations calling for culturally competent care (Office of Minority Health, 2001).

Existing reviews have examined cultural competency and related concepts within health care settings such as nursing [3] and mental health [4] as well as within health care systems [5]. Some reviews have focused on either provider outcomes [6] or patient/client outcomes [7] while others have examined specific health conditions such as diabetes [8].

These existing reviews highlight a lack of robust evidence pertaining to the relationship between cultural competency and improved provider/organizational behaviors or patient/client health outcomes. There is also a lack of consensus on the most effective ways of improving cultural competency [9] and continuing debate as to whether interventions to improve cultural competency can lead to a reduction in health disparities caused by racial/ethnic discrimination [10]. The aim of this systematic review of reviews is to gather and synthesize existing reviews of studies in the field to form a comprehensive understanding of the current evidence base that can guide future interventions and research in the area.

For this review of reviews, interventions to improve cultural competency are defined as those that aim to: improve the accessibility and effectiveness of health care for people from racial/ethnic minorities by increasing awareness, knowledge and skills of health care providers or patients as well as modifying policies and practices of organizations. These interventions may be focused at the health care provider-patient/client level (e.g. interpersonal interactions) or more broadly at the organizational level (e.g. integration cultural competency into policies, plans and processes). Interventions that meet this definition may also be referred to in this paper by other terms such as culturally appropriate care and multicultural education if these terms are utilized in specific reviews.

## Methods

### Search strategy

In November 2011 the following databases and electronic journal collections were searched from 2000 to 2011: Medline, Cinahl, Eric, PsycINFO, Proquest (Dissertation/Theses), Scopus and the Cochrane Systematic Review Database. Reference lists were hand-searched for other reviews. Key websites (i.e. http://www.diversityrx.org, http://www.nccc.georgetown.edu, http://www.minorityhealth.hhs.gov, http://www.ceh.org.au, http://www.hrsa.gov, http://www.nice.org.uk) were also searched. In July 2012 the search was updated to include recent studies published up to June 2012. See Additional file 1 for search terms used.

As cultural competency did not achieve popularity until the late 1990s and government policies mandating cultural competence did not occur until the early 2000s [11], a search timeframe of 2000–2012 was chosen.

### Inclusion and exclusion criteria

A review was considered eligible for inclusion if it met the following criteria: i) included quantitative, qualitative or mixed methods studies, ii) was written in English, iii) included studies involving: health care/service providers/practitioners/clinicians, health administrators, support staff and patients/clients/health service users, iv) included studies utilizing any strategies or interventions to improve cultural competency (e.g. training programs or workshops or educational courses), v) included studies involving intervention settings or services related to the health sector (e.g. hospitals, community health services, educational institutions teaching health related courses), vi) included studies that used one or more outcome measures at an individual level (e.g. survey), organizational level (e.g. programs) or system level (e.g. policies).

Reviews were excluded if they described cultural competency in other non-health settings (e.g. education system), were conducted prior to the year 2000, or did not contain a methods section that included information on: search strategy, number of included studies, and details of studies.

### Quality of the review and synthesis of results

Each review was critically appraised independently by two authors using the health-evidence.org tool for reviews [12]. This tool consists of ten questions to assess the quality of the review using commonly accepted evidence-informed principles. Reviews were given a quality assessment rating of weak, moderate or strong.

### Identification of reviews

**Figure 1:**
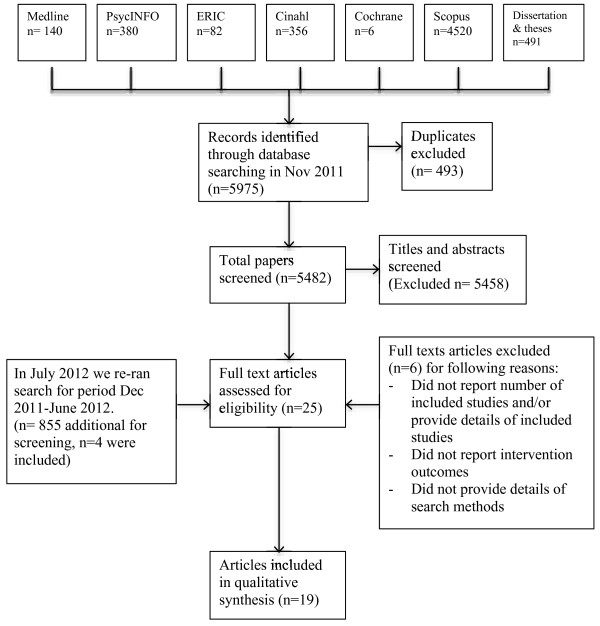
PRISMA flow diagram search process – initial search conducted December 2011.

The total search identified 6,830 results. Based on the inclusion and exclusion criteria, titles and abstracts were screened for eligibility by the first author. Full texts were retrieved for all reviews where inclusion was in doubt. To reduce the potential for bias in the screening process, the second author independently screened 10% of the total identified titles and also extracted data for 10% of the reviews that met the inclusion criteria. There was no difference in agreement between reviewers. See Figure 1: PRISMA flow diagram for a flow chart summary of the search and inclusion/exclusion process.

### Data extraction and analysis

Data extracted from the included reviews was entered into an Excel 2011 spreadsheet under the following headings: author(s), year of publication, health care setting/context, definition/concept/framework of cultural competence, method of review (e.g. database(s) searched), inclusion/exclusion criteria, number of included papers, types of papers included, study quality assessment, major findings, recommendations and quality/critical appraisal of review.

Data extracted from the reviews was descriptively analyzed using Excel 2011. Meta-analysis was not conducted due to the heterogeneity of the reviews and their included studies. Analysis focused on: types of interventions and study outcomes.

## Results

### Overview of reviews

Searching yielded a total of 6,830 titles, of which 19 met the inclusion criteria and were extracted for analysis (Table 1) [3-8,13-25]. The main reasons for exclusion were: articles were commentary or opinion pieces, articles were of primary studies, review articles examined cultural competency assessment tools and review articles but did not include any studies with interventions. Six review articles were excluded for not providing information on search strategy and details of included studies [26-31].

**Table 1:** Summary of 19 included reviews

**Author & year of publication**	**Health context**	**Definition of cultural competence**	**Sources (years of search)**	**Number of included papers**	**Type of papers**	**Outcomes**	**Was study quality assessed?**	**Major findings (review authors’ conclusions)**	**Review quality**
Anderson et al. 2003	Healthcare systems	Based on Cross et al. 1989 definition: ‘a set of congruent behaviors, attitudes and policies that come together in a system, agency, or among professionals and enable effective work in cross-culturally situations’	Medline, Eric, Soc Abs, SciSearch, Dissertation Abs, Soc Sci Abs, Mental Health Abs, Healthstar. English only. (1965–2001)	6	Intervention studies	1) Patient satisfaction, health status 2) utilization of health services	Yes	Could not determine the effectiveness of any of these interventions, because there were either too few comparative studies, or studies did not examine the outcome measures evaluated in this review: client satisfaction with care, improvements in health status, and inappropriate racial or ethnic differences in use of health services or in received and recommended treatment.	Moderate-strong
Beach et al. 2005	Health professionals (physicians and nurses). Most studies located in the United States.	Cultural competence has been defined as “the ability of individuals to establish effective interpersonal and working relationships that supersede cultural differences” (Cooper et al. 2002) by recognizing the importance of social and cultural influences on patients, considering how these factors interact, and devising interventions that take these issues into account (Betancourt et al. 2003).	Medline, Cochrane, Embase, EPOC, RDRB/CME, Cinahl (1980–2003)	34	RCTs, controlled, pre & post	1) Provider outcomes: knowledge, attitudes, skills 2) patient outcomes: satisfaction, behaviors, health status 3) cost effectiveness	No	Cultural competence training shows promise as a strategy for improving the knowledge, attitudes, and skills of health professionals. However, evidence that it improves patient adherence to therapy, health outcomes, and equity of services across racial and ethnic groups is lacking. It is difficult to conclude from the literature which types of training interventions are most effective on which types of outcomes. Also difficult to determine which types of knowledge, attitudes & skills are impacted by training.	Moderate- strong
Bhui et al. 2007	Mental health. All studies located in North America.	Aim of the paper is to develop a meaning of CC	Ingenta, Medline via Ovid, Medline via Pubmed, Medline Plus, Health Outcomes, HealthPromis, HSTAT, DocDat, National Research Register, NLM Gate- way, Cam, ReFer and Zetoc. (1985–2004)	9	No RCTs. Qualitative & quantitative papers	1) Provider outcomes 2) evaluations of implemented models of CC	No	There is limited evidence on the effectiveness of CC training and service delivery. Few studies published their teaching and learning methods. Only three studies used quantitative outcomes. One of these showed a change in attitudes and skills of staff following training. No studies investigated service user experiences and outcomes.	Moderate-strong
Chipps et al. 2008	Health professionals working in community-based rehabilitation including mental health and primary care. All studies located in North America.	“The ability to effectively provide services cross-culturally” (Diller 1999). Cultural competence training programs aim to increase “cultural awareness, knowledge, and skills leading to changes in staff (both clinical and administrative) behavior and patient-staff interactions” (Brach & Fraserirector 2000). Cultural competence includes the capability to identify, understand, and respect values and beliefs of others (Anderson et al. 2003).	CINAHL, Medline, Pubmed, PsycINFO, SABINET, Cochrane, Google, NEXUS, and unpublished abstracts (1985–2006)	5	RCTs, quasi-experimental, evaluation studies	1) Provider outcomes: cultural knowledge and attitudes, cultural competence, 2) patient health outcomes: satisfaction, behaviors, health status	Yes	Positive outcomes were reported for most training programs. Reviewed studies generally had small samples and poor design. 3 of the 5 studies reported on patient/client satisfaction.	Strong
Downing et al. 2011	Health care workers in Australia	Throughout this review, the term ‘indigenous cultural training’ will be used to describe training that is concerned with assisting health workers to provide health care that is accessible, meaningful and useful to indigenous/other minority groups in terms of their social, emotional and cultural wellbeing as well as physical health.	CINAHL PLUS, MEDLINE, Wiley InterScience, ATSIHealth and ProQuest.	9	Not reported	1) Provider outcomes: knowledge, attitudes, awareness,	No	There is scant evidence for the effectiveness of indigenous cultural training. The only study to assess knowledge and attitudes before and after training with a control group found no effect. Three studies also documented positive post-training reports but it is unclear if this relates to any change in practice as a result of the training. No information was available with which to assess systemic differences between the programs that did and did not produce (perceived) changes.	Moderate
Fisher et al. 2007	Health care provision to non-White racial and ethnic groups in the United States.	Cross’ definition for cultural competence. Definition of cultural leverage: a focused strategy for improving the health of racial and ethnic communities by using their cultural practices, products, philosophies, or environments as vehicles that facilitate behavior change of patients and practitioners.	Medline, Cochrane, Web of knowledge, The New York Academy of Medicine Grey Literature Report (1985–2006)	38 (35 unique studies)	RCTs, pre-post, controlled	1) Patient outcomes: health behaviors 2) access to health care system 3) provider: cultural competence	Yes	The interventions reviewed increased patients’ knowledge for self-care, decreased barriers to access, and improved providers’ cultural competence. Interventions using cultural leverage show promise in reducing health disparities, but more research is needed.	Moderate
Forsetlund et al. 2010	Health care for ethnic minorities. Most studies located in the United States.	To collect and summarise in a systematic and transparent manner the effect of interventions to improve health care services for ethnic minorities	Cochrane Library, MEDLINE, EMBASE, British Nursing Index, ISI Social Sciences/Science Citation Index (SSCI/SCI) and Research and Development Resource Base (RDRP).	19	Randomized controlled	Quality of health care services, use of health care services, patient health or the quality of life for patients.	Somewhat	Educational interventions and electronic reminders to physicians may in some contexts improve health care and health outcomes for minority patients. The quality of the evidence varied from low to very low. The quality of available evidence for the other interventions was too low to draw reliable conclusions.	Moderate-strong
Harun et al. 2012	Cancer care to ethnic minority women. All studies located in the United States.	Defines “patient-centred care”: involves integrating patient preferences and values to guide clinical decisions and management, and it is thought to facilitate improved patient satisfaction, communication with providers, safety, costs and efficiency in the health-care system.	Medline, PsycINFO, EMBASE and Cochrane	7	Randomized controlled, non-randomized, mixed-method experimental	Communication with health providers, decision-making, treatment adherence, general patient participation, treatment knowledge	Yes	Of the 37 selected studies, only 18 included valid outcome measures. Employing a combination of multiple strategies is more likely to be successful than single interventions. The impact of the interventions on participation was varied and effectiveness may hinge on a variety of factors, such as type of intervention and study population characteristics. Given the paucity of studies, it is difficult to draw conclusions about the effectiveness of the different interventions for this broad patient group.	Moderate-strong
Hawthorne et al. 2008	Community-based or hospital-based settings. Diabetes education for ethnic minority groups. Most studies located in the United States.	’Culturally appropriate’ health education is defined here as education that is tailored to the cultural or religious beliefs and linguistic skills of the community being approached, taking into account likely literacy skills (Overland 1993).	The Cochrane Library, MEDLINE, EMBASE, PsycINFO, CINAHL, ERIC, SIGLE and reference lists of article (prior to 2007).	11	RCTs	Patient: health status, behaviors, satisfaction, knowledge.	Yes	Culturally appropriate diabetes health education appears to have short-term effects on glycaemic control and knowledge of diabetes and healthy lifestyles. None of the studies were long-term, and so clinically important long-term outcomes could not be studied. No studies included an economic analysis.	Strong
Henderson et al. 2011	Chronic health conditions. Most studies located in the United States.	Culturally safe services were originally defined as those where there is no assault on a person’s identity caused by the fact that service delivery methods or processes are alien to the person’s culture (Ramsden 1990).	CINAHL, MEDLINE, Joanna Briggs Institute, Cochrane Library, Lippincott, Williams and Wilkins Collection, PubMed, ProQuest, Dissertations and Theses, and Google Scholar (1999–2009)	24	RCTs and controlled trials	1) Utilization of health services 2) patient outcomes: satisfaction, health behaviours, health status 3) provider outcomes: awareness, cultural competency	Yes	The review supported the use of trained bi-lingual health workers, who are culturally competent, as a major consideration in the development of an appropriate health service model for culturally and linguistically diverse communities. Four studies reviewed involved cultural competency training for healthcare providers and all 4 indicated that cultural competency training was beneficial. Nevertheless, the translation of cultural knowledge into practice remains problematic.	Moderate
Kehoe et al. 2003	Health care for ethnic minority groups. Most studies located in the United States.	CC involves the actual integration of congruent behaviors, attitudes and policies, within the delivery of health care in cross-cultural situations. (Office of Minority Health 2000)	Medline, Cinhahl (1980–2001)	14	RCTs, quasi-experimental	Patient outcomes: health status, health behaviors	Yes	A small number of studies demonstrated significantly improved outcomes for patients with diabetes mellitus, drug addictions, sexually transmitted infections and other health problems, after receiving culturally competent or relevant interventions. Few studies examined long-term effects of interventions on health outcomes.	Moderate
Kokko 2011	Nursing. Participants in the studies were from Australia, Denmark, Finland, Germany, Norway and Sweden.	Cultural competence is defined as a set of skills and behaviors that enable a nurse to work effectively within the cultural context of a client/patient (Leininger 2002, Papadopoulos 2006).	MEDLINE and Cumulative Index to Nursing and Allied Health Literature (CINAHL) databases (2000–2009)	7	Qualitative	1) Provider outcomes: cultural knowledge, personal growth, nursing student’s practice, preparedness for cultural competence in nursing	No	The results of the present study demonstrate that participating in overseas student exchange programs increased the nursing students’ preparedness to be culturally competent.	Weak-moderate
Lie et al. 2011	Health care professionals. Most studies located in the United States.	Not reported	MEDLINE/PubMed, ERIC, PsycINFO, CINAHL and Web of Science databases (1990–2010)	7	Intervention studies	Patient outcomes: satisfaction, behaviors, health status	Yes	Study quality was low to moderate. Effect size ranged from no effect to moderately beneficial. There is limited research showing a positive relationship between cultural competency training and improved patient outcomes.	Strong
Lu et al. 2012	Cancer screening involving Asian women. Most studies located in the United States.	Not reported	MEDLINE, EMBASE, Cochrane Database of Systematic Reviews, Cochrane CENTRAL Register of Controlled Trials, CINAHL, CancerLit, DARE Database of Reviews of Effects, PsycINFO, ABI Inform, ERIC, Social Sciences Abstracts, Sociological Abstracts, Health Technology Assessment Database (University of York), Proquest Dissertations and Theses, and KUUC Knowledge Utilization Database (University of Laval)	37	Randomized control trial (including cluster randomized trial, and randomized controlled crossover trial), non-equivalent control group, or prospective cohort.	Breast cancer screening, cervical cancer screening, and those studies targeting both breast cancer and cervical cancer screening	Yes	Our review found that intervention studies varied greatly by study population and geographic area. Therefore we could not arrive at a conclusive and generalizable conclusion on effectiveness of any one particular intervention. Only eighteen of the included studies reported effectiveness based on completion of mammograms or pap smear, either by self-report and/or verified through clinical record. While some studies demonstrated the effectiveness of certain intervention programs, the cost effectiveness and long-term sustainability of these programs remain questionable.	Moderate-strong
McQuilkin 2012	Nursing. Participants in the studies were mostly from the United States	Evidence of awareness of personal culture, values, beliefs, attitudes and behaviours; demonstrated ability to assess cross-cultural variations; and to effectively perform requisite skills needed to assess and communicate with individuals from other cultures (Cavillo et. al, 2009).	Health and Psychosocial Instruments, CINAHL Plus with Full Text, ERIC, Health Source: Nursing/Academic Edition, MEDLINE, PsycINFO, EBSCO, COCHRANE, CINAHL, reference lists from identified articles.	37 (16 interventions)	Case study, expert opinion, comparative descriptive, quantitative, systematic review	1) Increased self-awareness of their own values, attitudes, beliefs and behaviors that compose their culture, 2) increased skill in assessment and communication with persons from other cultures, and 3) ability to provide an assessment of transcultural differences	Yes	Findings demonstrated that international immersions provided optimal experiences to develop cultural competence alone, but more effective when combined with other strategies. International immersion experiences can increase student self-awareness, cross-cultural communication and assessment skills, and ability to assess cultural differences. The evaluation measures described in the literature were consistently student self-perception rather than observed development of the student’s cultural competence.	Moderate
Pearson et al. 2006	Nursing	Definition: “the ability of systems to provide care to patients with diverse values, beliefs and behaviors, including tailoring delivery to meet patients’ social, cultural and linguistic needs” (Betancourt et al. 2002)	CINAHL, Medline, Current Contents, the Database of Abstracts of Reviews of Effectiveness, The Cochrane Library, PsycINFO, Embase, Sociological Abstracts, Econ lit, ABI/Inform, ERIC and PubMed. The search for unpublished literature used Dissertation Abstracts International. (prior to 2005)	19	Quantitative, qualitative, reviews	1) Patients: health status, satisfaction 2) nurses: 3) organisations 4) systems	Yes	The results identified a number of processes that would contribute to the development of a culturally competent workforce. Appropriate and competent linguistic services, and intercultural staff training and education, were identified as key findings in this review.	Moderate -strong
Smith et al. 2006	Mental health professions. All studies were located in the United States.	Not reported	Dissertation Abstracts, ERIC, HealthSTAR, Medline, Mental Health Abstracts, Programme Applique’ a’ la Selection et a’ la Compilation Automatiques de la Litte’rature, PsycINFO, Social Sciences Abstracts, Social SciSearch, Sociological Abstracts via SocioFile, and Social Work Abstracts (1973–2002)	Meta-analysis 2 n = 37	Outcome studies	Meta-analysis 2- provider outcomes: multicultural counseling competence, racial identity, racial prejudice, client-counselor relationship	Yes	Multicultural education interventions were typically associated with positive outcomes across a wide variety of participant and study characteristics. Multicultural education interventions that were explicitly based on theory and research yielded outcomes nearly twice as beneficial as those that were not.	Moderate-strong
Sumlin & Garcia 2012	Diabetes management involving African American women in the United States	Cultural competence, or tailoring, is defined as “the process of creating culturally sensitive interventions, often involving the adaptation of existing materials and programs for racial/ethnic subpopulation”.	PubMed, Cumulative Index to Nursing and Allied Health Literature, the Cochrane Review database, and The Diabetes Educator journal index.	15	RCTs and quasi-experimental designs	Dietary outcomes, weight loss, changes in metabolic control (A1C), lipids, blood pressure, and cholesterol	No	Of the 15 studies, 6 showed significant improvements in food practices, and 8 showed significant improvements in glycaemic control as a result of the interventions. It is not clear what components of the 15 interventions were most effective. Most studies did not report the duration of the sessions, thereby making comparison of “intervention dose” impossible. In addition, variations across the studies in content and methods used do not point to specific recommendations for clinicians or educators to adopt or avoid.	Moderate-strong
Whittemore 2007	Diabetes management involving Hispanic adults in the United States.	Not reported	CINAHL, Medline, PsycINFO (1990–2006)	11	RCTs, pre-post design	Patient outcomes: clinical, behavioral and knowledge	No	The majority of studies in this review reported significant improvements in select clinical outcomes, behavioral outcomes, or diabetes- related knowledge.	Moderate-strong

The majority of reviews (n = 15) were published between 2007 and 2012. Reviews focused on a range of health care settings/contexts, including: health professionals, community rehabilitation, nursing and health systems. A range of study designs were included in the reviews, including randomized controlled trials, pre and post designs as well as qualitative studies. Most reviews provided a definition of cultural competency or related concept. The number of studies included in each review varied between 5 and 38. Smith et al.’s [19] review consisted of two meta-analyses, of which only the second meta-analysis (n = 37) met the inclusion criteria. (The first meta-analysis consisted of retrospective survey studies that did not report outcome measures.) Thirteen reviews assessed the quality of studies using critical appraisal tools such as the Oxford Centre for Evidence Based Medicine [13].

### Interventions to improve cultural competency

Types of interventions to improve cultural competency included in the reviews were: training/workshops/programs for health practitioners (e.g. doctors, nurses and community health workers), culturally specific/tailored education or programs for patient/clients, interpreter services, peer education, patient navigators and exchange programs.

Seven of the 19 reviews focused solely on healthcare provider cultural competency interventions [6,7,13,14,17,19,23] whilst six reviews examined only culturally competency interventions aimed at patients/clients [8,18,20-22,24]. Three reviews included studies that examined organizational level interventions such as culturally adapting health programs for patients and employment of bilingual community health workers [5,15,16]. One review focused primarily on interventions directed at health care personnel and/or organizations, although interventions targeting both health personnel and patients were also included [25]. Another review looked at the structures and processes that support the development of culturally competent practices [3]. Evaluated models of cultural competence in mental health were reviewed by Bhui et al. [4].

### Study outcomes

There were three main categories of study outcomes amongst the reviews: provider-related outcomes, patient/client-related outcomes and outcomes related to health service access and utilization. Evaluations of implemented models of cultural competency [4] and cost-effectiveness [6] were also examined.

### Provider outcomes

Measured provider outcomes focused on knowledge, attitudes and skills related to cultural competency. In Beach et al.’s [6] review, knowledge refers to information about general cultural concepts such as the impact of culture on the patient-provider encounter or culture-specific knowledge such as traditional cultural practices. Attitude outcomes measured by studies included cultural self-efficacy (assessing learner confidence of knowledge and skills in relation to ethnic minority patients), attitudes towards community health issues, and interest in learning about patient and family backgrounds [6]. Skills included communication skills or use of treatment plan. In contrast, Smith et al.’s [19] review used multicultural counseling competence as their main outcome measure while Kokko [17] included studies examined nursing students’ cultural knowledge, personal growth and nursing practice.

### Patient/client outcomes

There were a variety of patient/client outcomes reported, including physiological outcomes such as blood glucose, weight and blood pressure [8] as well as outcomes such as patient satisfaction and trust [7], knowledge of cancer screening and knowledge of health conditions [15]. Behavioral outcomes such as dietary and exercise behaviors were also examined in three reviews [15,18,21]. Other reviews looked at primarily patient-focused interventions to improve breast and cervical cancer screening among women [24] and to improve participation in cancer treatment processes [22].

### Health service access and utilization outcomes

Outcomes related to health service access and utilization included use of bilingual community health workers, interpreters, and patient navigators. These interventions were designed to influence individuals’ ability to access the resources of health care organizations by bridging the cultures of the organizations and those of the target communities [15].

Cost-effectiveness of interventions was considered in three reviews [6,8,24]. Beach et al. [6] noted that only 4 of 34 studies included in their review addressed the costs of cultural competence training. No studies in Hawthorne et al.’s [16] review measured the cost effectiveness of their interventions, although some included a rough estimate of costs. Two studies in Lu et al.’s [24] review reported cost information. These reviews noted this as an important limitation of studies they examined.

### Major findings of reviews

#### Provider related outcomes

Six of the eight reviews that examined healthcare provider interventions found some evidence of improvement in provider outcomes such as knowledge, skills and attitudes in relation to cultural competency [6,15-17,19,23].

#### Patient/client related outcomes

Seven of the nine reviews that examined patient/client-related outcomes generally found evidence of some improvement in health outcomes. Hawthorne et al.’s [8] review of culturally appropriate diabetes health education found short-term effects (up to one year) on glycemic control and knowledge of diabetes and healthy lifestyles. However, long-term effects (one year or more) were not examined by any studies. Whittemore [20] also reviewed culturally appropriate interventions in relation to diabetes, but for Hispanic populations only, finding evidence of significant improvements in selected clinical outcomes, behavioral outcomes and diabetes-related knowledge in the majority of studies. Sumlin & Garcia [21] found significant improvement in food practices and glycemic control amongst African American women with Type 2 diabetes following use of culturally competent food-related interventions. Kehoe et al.’s [18] review also found that culturally relevant interventions improved patient/client outcomes for conditions such as diabetes and drug addiction. Lie et al. [7] found a positive relationship between cultural competency training and improved patient/client outcomes. Chipps et al.’s [13] review included three studies measuring patient/client satisfaction, with only one of these three studies finding increased satisfaction of clients with their counselors. Reviews by Harun et al. [22] and Lu et al. [24] found mixed results, and hence were unable to draw generalizable conclusions in relation to patient participation in treatment and cancer screening, respectively.

#### Outcomes related to access and utilization of health services

Four of five reviews that included studies related to health service outcomes found some evidence of improvement. Fisher et al. [15] reviewed a range of interventions to narrow racial disparities in primary and tertiary health care settings, grouped into three categories: patient behavioral change, access to care, and health care organization innovation. They found that interventions using culturally specific patient navigators and community health workers were among the most successful. Henderson et al. [16] reviewed a range of culturally appropriate interventions to manage chronic disease among racial/ethnic minorities, also finding support for the use of trained bilingual health workers to promote greater uptake of disease prevention strategies. From a healthcare systems perspective, Anderson et al. [5] found a lack of both quantity and quality of studies focused on improving cultural competency. Pearson et al. [3] found that appropriate and competent linguistic services and intercultural staff training and education were key in developing effective culturally competent practices in nursing. Forsetlund et al. [25] found that education interventions and electronic reminders to physicians may improve health care and health outcomes for minority patients. However, the quality of evidence for these interventions was graded as low to very low.

#### Other outcomes

Bhui et al.’s [4] review included studies that evaluated implemented models of cultural competence; essentially organizational approaches. They concluded that culturally competent care and services at the organizational level is addressed in different ways depending on the local context, for example managed care and insurance based service models in the United States may not to translatable in settings where services are dependent on government funds.

### Quality of studies within reviews

The majority of reviews noted methodological limitations of studies. This limited conclusive statements about the effectiveness of interventions to increase cultural competency. The main methodological criticisms of the studies by the reviews were: small samples [13], poor methodological rigor [7,13,15], no or few long-term studies [8,18], no economic analysis of interventions [6,8], reliance on self-report measures [19], lack of detail about interventions [7,19], lack of patient outcome measures [4-6,15] and lack of objective provider measures related to change in practice [14,17].

Some reviews reported the quality/strength of evidence supporting the outcomes measured [5-8,15,25]. For example, Beach et al. [6] graded the strength of evidence for each outcome type based on its quality, quantity, and consistency (grades A – D). In their review, evidence of impacts on provider knowledge were graded A compared with provider attitudes which were graded B.

### Recommendations of reviews

Twelve of the nineteen reviews concluded that further research (e.g. more rigorous trials and evaluations) was required to determine the effectiveness of interventions to improve cultural competency for providers and patients/clients. The reviews found that many of the studies were difficult to compare as different frameworks of cultural competency were used and studies often lacked a standardized and validated instrument to measure cultural competence [6]. Most reviews concluded that training had positive impacts on provider outcomes. However, it was difficult to determine exactly what types of training interventions were most effective in relation to particular outcomes [6,13,19]. A need for research into long-term outcomes [8,18] was identified along with the need to consider other factors that facilitate cultural competency, such as links with community organizations [3,15]. It was also recommended that cost-effectiveness be assessed [8,24].

### Limitations of reviews

Some of the reviews focused on one type of intervention such as diabetes education for patient outcomes [8] or health provider cultural competency training [14]; one type of study outcome such as patient outcomes [7]; one type of study design such as randomized controlled trials [25]; or a particular study population such as Hispanics [20], Asian women [24] or nurses [17]. Although it may be more feasible for a review to focus on a particular group of health providers or type of health care setting, it limits generalizability and applicability of the findings. Many studies were heterogeneous in outcome and interventions, making statistical synthesis and analysis difficult [13]. According to Smith et al. [19], their meta-analysis was limited by: studies with single-group pre-to post-test assessments (e.g. [32,33]), studies rarely reporting disaggregated data, and predominantly self-report measures (e.g. [34,35]).

It is also difficult to determine the extent to which knowledge and skills learnt from training/programs are translated into practice and how they impact on patient/client outcomes [5,17]. Provider outcomes determined by self-report are subject to multiple threats to internal validity [36] and hence limit the conclusions made regarding impact on provider practice [19] and ultimately on patient outcomes.

The literature includes a diverse range of populations (e.g. African American, Hispanic/Latino, and Asian), health care settings (e.g. community centers, hospitals and academic medical centers) and interventions (e.g. culturally tailored programs for particular racial/ethnic groups and provider training). However, the majority of studies were based in the United States. Two reviews limited their included studies to only those conducted in the United States [15,21].

### Heterogeneity of reviews and studies

Meta-analysis was not conducted in this review of reviews due to the heterogeneity of the reviews and their included studies. Intervention effects were also difficult to determine as only some reviews described outcomes in terms of statistical significance and effect sizes [5,6,8,13,19]. Some reviews noted that studies rarely provided sufficient information on the curriculum or format, or details of the providers involved (e.g. age, race, gender, prior training) making it difficult to conclude from studies which types of training interventions were most effective for which groups and in producing which particular outcomes [6,7].

### Critical appraisal of reviews

All reviews were critically appraised by two authors using the health-evidence.org tool for reviews [12] and companion tool dictionary [37]. This process was reliant on the author’s prior knowledge and experience of the topic, research principles and study design methods. There were minor disagreements between authors and consensus was reached through discussion. Reviews were predominantly of moderate-strong quality (overall assessment of review quality is included in Table 1).

### Design of reviews

All reviews had a clearly focused question in relation to the population, intervention and outcomes. Appropriate inclusion criteria to select primary studies were used by the majority of reviews. The majority of reviews described comprehensive search strategies, although some were slightly limited in scope [4,14,15,20]. For example, Kehoe et al. [18]’s search strategy consisted of only two electronic databases. The number of years covered by the search strategies was 20 years or more by the majority of reviews. Two reviews [16,17] covered 10–11 years, one review searched between 2005–2011 [23], and one review [14] did not provide this information.

### Methodological rigor of reviews

The methodological rigor of studies was identified and described in thirteen reviews [3,5,7,8,13,15,16,18,19,22-25]. The methodological rigor of primary studies using an assessment tool/scale was conducted by all these reviews except for two [18,19]. Nine reviews reported the use of two or more reviewers to assess each study for methodological quality [6-8,13,15-17,22,24]. Most reviews used appropriate methods for combining and comparing results across studies. However, Pearson et al.’s [3] results were not well presented.

## Discussion

This systematic review of reviews has identified a number of key issues and limitations in what is currently known about interventions to improve cultural competency within healthcare. There was considerable heterogeneity amongst the reviews in relation to interventions used, patient populations, health provider populations, health contexts/settings as well as processes and outcomes of care. This reflects the complexity of the area and its translation to practice and research. Overall, positive effects were reported by most reviews, particularly in relation to provider outcomes. However, it remains unknown exactly what types of interventions are most effective, for whom, in what context, and why.

Reviews that compared different types of interventions, e.g. Henderson et al. [16] and Fisher et al. [15], found that the use of culturally trained health workers was the most effective. However, rather than being comparable, many of the primary studies in these reviews were a mixture of study designs focused on various interventions.

The included reviews were generally difficult to compare as different definitions and frameworks of cultural competency or related concepts were used. Some reviews did not provide a definition [4,7,19,20,24]. The lack of uniformity in terminology and definition reflect the many variations of terms and definitions used in relation to cultural competency at present. This is likely a key contributing factor to the lack of consensus on the best ways to develop, implement and evaluate cultural competency interventions. Developing such consensus regarding terminology and definitions, with a view to improving evidence of effective cultural competency interventions is thus an important area of future work both theoretically and empirically.

Mixed findings were found by two reviews [22,24]. In their review of breast and cancer screening among Asian women, Lu et al. [24] determined that the effectiveness of interventions to promote screening depended on factors such as the type of intervention, methods of program delivery, study setting and ethnic population. Harun et al.’s [22] review of interventions to improve participation in treatment found that the impact of these interventions was varied amongst the seven included studies. Both reviews found that patterns of intervention design and results of effectiveness were heterogeneous, therefore it was difficult to generalize the effectiveness of particular interventions for particular patient/client groups.

### Organizational context

Cross-cultural interactions are likely structured and shaped by the worldviews and past experiences of not only the staff and clients but also the culture of the organization, which is embedded in and produced by policy frameworks, organizational arrangements and physical settings of the organization [38]. Interventions to improve cultural competency need to consider the individual and organizational contexts and the interplay between them. Training programs may need to be tailored to particular groups, for example physicians would need particular knowledge and skills specific to their clinical tasks that would be inapplicable for reception staff.

It is likely that cultural competency training as a stand-alone strategy is insufficient to improve patient outcomes without concurrent systemic and organizational changes [7,9,39]. Embedding cultural competency in organizational policy documents such as position statements and strategic plans are more likely to result in sustained change within organizations. There should be a commitment among the leadership of the organization and embedded key performance indicators supported by allocated resources.

There is some evidence of a relationship between the cultural competence of health practitioners and the cultural competence of organizations [39,40]. Providers may be influenced by their organization’s commitment and actions in relation to cultural diversity and vice versa. A study found that providers with attitudes reflecting greater cultural motivation to learn were more likely to work in clinics with more culturally diverse staff and those offering cultural training and culturally adapted patient education materials [40].

In recognition that different components of the health system influence health outcomes, some models of cultural competency advocate a multi-level approach [2,41]. Although some studies have shown that culturally competent practices among organizations are adopted to varying degrees [42,43], more research is needed in this area. Grol et al. [44] found that empirical evidence of the effectiveness and feasibility of most theoretical approaches to produce change in health care was limited. Dreachslin et al. [45] found a paucity of research on organizational behaviour in the healthcare and general management literature. A more recent review by Parmelli et al. [46] showed limited available evidence regarding effective strategies to change organizational culture in health care. Barriers and incentives to organizational change should be considered when designing and implementing an intervention to increase the likelihood of success and sustained change [47,48]. Issues related to organizational readiness for change and innovation also require consideration before implementing organizational cultural competency interventions [49]. Understanding the reasons for adoption and spread of innovation can assist with addressing the difficulties of organizational change [50]. Planning and implementation of cultural competency interventions should acknowledge the interaction between an intervention and the setting. Organizational cultural competence involves an understanding of the strengths and weaknesses of the health care organization and the unique needs of the people it serves.

### Beyond self-assessment

Self-assessment was the most common approach to assessing cultural competency, which is a subjective measure subject to a range of biases [36]. The assessment tools were mostly process and survey tools, including patient satisfaction and provider self-assessment questionnaires as well as self-administered organizational checklists. Many of these tools have not been validated [51]. Self-rating at the individual level may be affected by the respondents’ level of cultural awareness and is subject to biases such as social desirability [52]. Broader organizational and systemic approaches to cultural competency should consider assessments of cultural competency that include objective measures such as document review [52]. Moving beyond self-assessment is a necessary step towards developing a stronger evidence base for the use of cultural competency related interventions to improve patient/client health outcomes. In addition, more research is needed to determine how well both individual-level and organizational-level guidelines for cultural competency are followed by those directly involved in service delivery [53].

### Broader issues of culture, racism and privilege

Academics have asked whether cultural competency can be achieved without focusing on issues related to racism and white privilege [54,55]. Concepts related to racism, bias and discrimination were noted in some reviews [3,5,6,14,15,23], although none were measured as outcomes in studies. Two of the 34 studies in Beach et al.’s (2005) review included mention of these concepts in their education content. Factors such as structural inequalities and racism may have a greater impact on health disparities between particular groups than cultural differences [56].

Self-reflection and awareness of one’s professional and personal culture is an important component of cultural competency [57]. Of the seven reviews that focused on provider outcomes, four discussed these concepts [13,14,17,23]. This is despite these self-reflexive elements being critical to cultural competency. Cultural awareness alone is inadequate for addressing the effects of structural and interpersonal racism on health disparities. Cultural awareness training has been criticized for increasing stereotyping and reinforcing essentialist racial identities [58]. Reflexive antiracism training is a promising alternative to cultural awareness training that reflects upon the sources and impacts of racism on society whilst avoiding essentialism and negative emotional reactions associated with White guilt [59].

There is a tendency within healthcare to equate culture with essentialized notions of race and ethnicity, which can lead to practices that separate culture from its social, economic and political context [10]. Narrow conceptualizations of culture and identity may limit the effectiveness of particular approaches, and a focus on specific cultural information may inadvertently promote stereotyping. Care must also be taken to avoid over-focusing on ‘culture’. Although cultural differences may worsen the problem of differential access and discrimination, broader factors such as poor education and poverty may play a greater role in the poorer health outcomes of some individuals and groups in the community [60]. However income and race/ethnicity as risk factors for health disparities can overlap and discrimination is often a driver of socio-economic disparities [61].

### Limitations

A limitation of this review is that there may be primary studies in the field that are not included by existing reviews in the literature as this was a review of reviews rather than a review of primary studies. There were only 22 primary studies cited by more than one review. This is likely due to the relatively narrow focus of some reviews e.g., by health condition, minority groups or type of outcome (i.e. patient or practitioner). Another limitation is that only reviews published in English were included. Given that the timeframe for this review was 2000–2012 it is possible that reviews published prior to 2000 were not included. However, cultural competency did not achieve popularity until the late 1990s and government policies mandating cultural competence did not occur until the early 2000s [11].

The search strategy could have been improved by adding more patient-related terms (e.g. migrant and refugee) and applying a more sensitive search filter for systematic reviews. It is possible that some reviews were excluded as a result, however it is unlikely that the overall findings would be significantly different as this paper includes reviews from various health care contexts and health care provider and patient populations as well as different types of studies and cultural competency interventions.

It is difficult to make a general statement about the strength of the effect of interventions as the reviews assessed their studies differently. Different critical appraisal tools were used by authors and a majority of reviews noted methodological limitations of studies in their reviews, which limited their ability to make conclusive statements about the effectiveness of interventions. In order to determine the strength of the effect, an assessment of all primary studies of the included reviews using a single critical appraisal tool to determine the effectiveness of their interventions and an assessment of quality and bias of each individual study is required. However this is beyond the scope of this paper.

## Conclusion

This systematic review of reviews of interventions to improve cultural competency within healthcare settings has synthesized all recent reviews in order to improve our understanding of the current evidence base and guide future research in this area. The majority of reviews found moderate evidence of improvement in provider outcomes and health care access and utilization outcomes. However, there was weaker evidence for improvements in patient/client outcomes.

This review has highlighted the breadth and complexity of research in this area as well as the popularity of this area as shown by the number of published reviews found during the period January 2000-June 2012, and particularly from 2007 onwards. Despite this popularity, it is clear that the evidence base is relatively weak, and there continues to be uncertainty in the field. First, there is no uniform definition or framework of cultural competence that is accepted across the spectrum of health contexts/settings either within or between countries. Many terms are used interchangeably with cultural competency (e.g. cultural safety, cultural awareness, cultural responsiveness). Second, there are many potential outcomes from cultural competency interventions, as indicated by the variety of measures utilized in reviews, but very few validated tools to assess cultural competency in the published literature [51,62]. Third, a lack of methodological rigor is common amongst the studies included in reviews. Moreover, many of the studies rely on self-report, which is subject to a range of biases, while objective evidence of intervention effectiveness was rare.

Future reviews should be explicit about their definition or framework of cultural competency and what constitutes a culturally competent intervention, whether at the individual-level, organizational-level or systemic-level. Reviews should also examine multiple outcomes at all three levels where possible due to the multi-dimensional nature of cultural competency interventions and the complexities in translating cultural competency into practice. Further development and assessment of organizational cultural competency models and assessment tools is needed [63].

Multi-level interventions should consider the different contexts (e.g. government policy vs. community issues) and cultures (e.g. individual vs. organizational) that can affect the implementation and success of interventions to improve cultural competency. Issues related to organizational change and understanding the mechanisms by which health innovations are adopted should also be taken into account. There is also need for research to examine the time and resources required to implement interventions in addition to identifying the most feasible and effective approaches [6]. This is particularly important for organizational or systemic approaches where cost-benefit/effectiveness is an important consideration.

## Competing interests

The authors declare that they have no competing interests.

## Authors’ contributions

All authors developed the idea for the systematic review and contributed to the concept and design. MT conducted the searching and drafted the tables and figures. All authors contributed to the writing of the manuscript and read and approved the final manuscript.

## Pre-publication history

The pre-publication history for this paper can be accessed here:

http://www.biomedcentral.com/1472-6963/14/99/prepub

## Supplementary Material

Additional file 1 Search terms used in search strategy. Click here for file
