# Supplementary material for: Interventions to improve cultural competency in healthcare: a systematic review of reviews
Source: BMC Health Serv Res. 2014 Mar 3;14:99. doi: 10.1186/1472-6963-14-99 (PMC3946184; doi:10.1186/1472-6963-14-99)
Supplement: Additional file 1 — Search terms used in search strategy. [file 1472-6963-14-99-S1.doc]

**Search terms**

Medline search strategy (adapted for other databases)

1. Cultur* adj (competenc* OR diverst* OR appropriat* OR safety OR respect OR responsiveness OR sensitiv* OR understanding OR knowledge OR expertise OR skill*)
2. Transcultural
3. Multicultural
4. Cross-cultur*
5. Cultural competency (MESH)
6. 1-5/OR
7. Educat*
8. Train*
9. Program*
10. Curricul*
11. Profession*
12. Course*
13. Intervention
14. Session
15. Workshop
16. Skill*
17. Instruct*
18. Program evaluation (MESH)
19. 6-17/OR
20. Provider
21. Practitioner
22. Professional
23. Physician
24. Doctor
25. Clinician
26. Primary health care (MESH)
27. Health Personnel (MESH)
28. 14-27/OR
29. Review
30. 6 AND 19 AND 28 AND 29
